# Association Between Oral and Pharyngeal Functions in Patients in Medical‐Dental Cooperation at an Acute Hospital

**DOI:** 10.1111/joor.70176

**Published:** 2026-03-08

**Authors:** Ryo Tagaino, Kana Saijo, Naru Shiraishi, Takamasa Komiyama, Kuniyuki Izumita, Takuma Hisaoka, Ai Hirano, Jun Ota, Yukio Katori, Toru Ogawa, Keiichi Sasaki, Nobuhiro Yoda, Hiroshi Egusa, Shigeto Koyama

**Affiliations:** ^1^ Maxillofacial Prosthetics Clinic Tohoku University Hospital Sendai Japan; ^2^ The Center for Dysphagia Tohoku University Hospital Sendai Japan; ^3^ Division of Molecular and Regenerative Prosthodontics Tohoku University Graduate School of Dentistry Sendai Japan; ^4^ Division of Advanced Prosthetic Dentistry Tohoku University Graduate School of Dentistry Sendai Japan; ^5^ Division of Aging and Geriatric Dentistry Tohoku University Graduate School of Dentistry Sendai Japan; ^6^ Department of Dental Informatics and Radiology Tohoku University Graduate School of Dentistry Sendai Japan; ^7^ Department of Otolaryngology‐Head and Neck Surgery Tohoku University Graduate School of Medicine Sendai Japan; ^8^ Division of Comprehensive Dentistry Tohoku University Hospital Sendai Japan; ^9^ Miyagi University Sendai Japan

**Keywords:** acute hospital, dysphagia, medical‐dental cooperation, oral function

## Abstract

**Background:**

Recent studies have focused on oral frailty and hypofunction, and their relationship with swallowing function and dysphagia. These play especially important roles in patients with dysphagia in medical‐dental cooperation at acute hospitals.

**Objective:**

This study aimed to assess oral frailty, in terms of nutritional intake by evaluating oral and swallowing functions simultaneously, in patients consulting at an acute hospital.

**Methods:**

This cross‐sectional study was conducted at the Center for Dysphagia of Tohoku University Hospital and involved a comprehensive survey of 183 patients. Their oral function and oral status were evaluated, which included factors such as poor oral hygiene, oral dryness, tongue‐lip motor function, tongue pressure, and masticatory performance. Additionally, swallowing status was evaluated using various methods, including a questionnaire related to swallowing, Hyodo–Komagane score, modified water‐swallowing, and repetitive saliva swallowing tests. This thorough approach ensured the validity and reliability of our findings.

**Results:**

Approximately 60% of patients had head and neck tumours, degenerative diseases, and muscular diseases. Therefore, a high prevalence of oral hypofunction was observed. Furthermore, since 41.5% of patients had limited tongue movement, a characteristic correlation existed between the oral diadochokinesis and Hyodo–Komagane score.

**Conclusion:**

These results suggest that oral dryness score, oral diadochokinesis, and tongue pressure are associated with oral hypofunction and dysphagia. Additionally, patients with hypofunction and decreased tongue pressure may have hypopharyngeal residuals. The medical‐dental cooperation in an acute hospital allows for the simultaneous assessment of oral and swallowing functions. This suggests a potential to contribute to the practice of safe oral intake and appropriate rehabilitation.

## Graphical abstract

This cross‐sectional study evaluated the oral and swallowing function of 183 patients at the Center for Dysphagia of Tohoku University Hospital. Results indicated that oral dryness, oral diadochokinesis, and tongue pressure are associated with dysphagia; importantly, patients with hypofunction and decreased tongue pressure may have hypopharyngeal residuals.

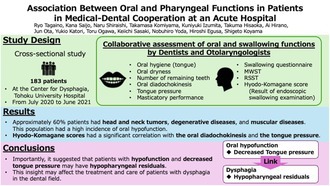

## 1 Introduction

Recently, the focus has been on oral hypofunction because it affects eating and swallowing functions as well as the whole body [1, 2]. Oral hypofunction is related to pharyngeal function and leads to eating and swallowing disorders, such as dysphagia [3, 4, 5]. Particularly, in patients with head and neck tumours, oral functions and eating conditions decline after surgery [6]. However, very few studies have investigated the relationship between oral and pharyngeal functions simultaneously in patients with dysphagia. Therefore, it is unclear as to which oral function and how decline in oral function affects pharyngeal function and eating ability.

Chewing and swallowing are closely related; chewing behaviour is the relationship between bolus transport and swallowing initiation [7]. A relationship between poor oral function and dysphagia, and the effects of modulation of masticatory movement on swallowing movements have also been reported [8, 9, 10, 11]. Proper bolus formation requires sufficient bite force, mastication, tongue movement, saliva flow and others. This proper bolus passes smoothly through the pharyngeal stage. Stage II transport, which occurs during mastication, is unconscious and smoothly transfers the bolus from the oral cavity to the pharynx. An appropriate oral environment and sufficient oral ability are necessary in both the oral and pharyngeal stages. Thus, to assess oral frailty based on nutritional intake from the oral cavity, it is important to evaluate both oral and pharyngeal functions. In particular, patients with head and neck tumours or those who have undergone surgery show a rapid decline in mastication and swallowing function, which leads to eating dysfunction or dysphagia [6]. In patients with head and neck tumours, it is vital to recover or improve eating and articulatory functions, because these improvements have nutritional and psychological effects that lead to an improved quality of life. However, few reports have linked feeding methods and oral and pharyngeal functions with nutritional status.

A ‘Center for Dysphagia’ was established at Tohoku University Hospital in July 2019 [12]. Here, we adopted a comprehensive approach as dysphagia intervention, allowing for the simultaneous evaluation of oral and pharyngeal functions. This medical‐dental cooperation can clarify the relationship between oral and pharyngeal functions and overall systemic health. In this study, we conducted a comprehensive survey of patients with dysphagia. The patients' oral function was thoroughly evaluated, and the relationship between oral status and swallowing function was explored. This study aimed to determine the oral function associated with swallowing function in these patients, as the finding would be an important factor in dental rehabilitation for patients with dysphagia.

## 2 Materials and Methods

### 2.1 Participants

At the Tohoku University Hospital, patients with dysphagia were referred to the Center for Dysphagia. If patients had any complications in the oral condition or problems with oral intake, a series of oral function assessments are conducted by the attending dentists. We included 183 patients who visited during the 1‐year period from July 2020 to June 2021. We excluded patients who opted out (notified and disclosed study information), who refused, and who were judged as difficult to participate. This cross‐sectional study was approved by the Ethics Committee of Tohoku University Graduate School of Dentistry (approval number: 38677, committee number: 11000400), and opt‐out information was posted on the website of the Division of Advanced Prosthetic Dentistry, Tohoku University Graduate School of Dentistry.

### 2.2 Collection of Patients' Information and Assessments

Patient information such as age, sex, primary disease, and use of supplementary nutrition was obtained from dentists' interviews and medical records. Four experienced dentists performed oral function examinations and three otolaryngologists performed endoscopic swallowing examinations.

Eichner's classification was based on the status of the four remaining occlusal support areas of the upper and lower jaws by the molars and premolars, and was classified into three: (A) with four occlusal support areas, (B) with reduced occlusal support areas, and (C) with no occlusal support areas. The need for removable dentures was classified into three categories: no need for removable dentures, removable dentures in use, and removable dentures necessary but not in use. The Simplified Oral Hygiene Index (OHI‐S) was used to assess oral hygiene. Nutritional management methods were categorised as oral intake alone, tube feeding alone, and combined oral intake and tube feeding.

Examinations for oral hypofunction were based on a position paper defined by the Japanese Society of Gerodontology [13]. Oral hypofunction can be diagnosed according to the degree of oral function and oral frailty. The diagnostic criteria for oral hypofunction were poor oral hygiene, oral dryness, reduced occlusal force (loss of the number of natural teeth), decreased tongue‐lip motor function, decreased tongue pressure, decreased masticatory function, and deterioration of swallowing function [13]. Poor oral hygiene was evaluated based on the tongue coating index, with a threshold defined as > 50%. Oral dryness was measured using an oral moisture checker Mucus (LIFE Co. Ltd., Saitama, Japan), where a score of < 27.0 indicated dryness. Tongue‐lip motor function was measured as the oral diadochokinetic rate (ODK) during the repeated pronunciation of each word ‘/pa/, /ta/, and /ka/’ using KENKOUKUN handy (Nippon Dental Shoji Co. Ltd., Tokyo, Japan). And poor tongue‐lip motor function was defined as < 6.0 times/s. Tongue pressure was measured using a tongue pressure test instrument TMP‐02 (JMS Co. Ltd. Tokyo, Japan). And poor tongue pressure was defined as < 30 kPa. Masticatory function was measured using a masticatory ability test Gluco sensor GS‐II (GC Co., Tokyo, Japan), with a value of < 100 mg/dL representing poor function. Finally, swallowing functions were evaluated using either the eating assessment tool (EAT)‐10 [14] or Seirei swallowing questionnaire [15]. Criteria for impairment were an EAT‐10 total score of ≥ 3 or a score of ≥ 1 on A group item of Seirei swallowing questionnaire. The oral hypofunction was diagnosed when three or more of the seven assessment items fall below each criterion.

Hyodo–Komagane scoring methods [16, 17], modified water swallowing test (MWST) and repetitive saliva swallowing test (RSST) were used for swallowing tests [18].

#### 2.2.1 Hyodo–Komagane Scoring Methods

To diagnose dysphagia, pharyngeal function was determined using the Hyodo–Komagane scoring method based on endoscopic swallowing examinations. This method comprised four parameters: (1) salivary pooling at the vallecula and piriform sinus, (2) glottal closure reflex induction by touching the epiglottis or arytenoid with the endoscope, (3) swallowing reflex initiation assessed by white‐out timing, and (4) pharyngeal clearance after swallowing blue‐dyed water or test jelly, each rated on a 4‐point scale from 0 to 3. The total score was calculated as the Hyodo–Komagane score [16, 17]. The Hyodo–Komagane scoring method was performed by experienced otolaryngologists.

The MWST was used to screen for swallowing function. In this test, patients were instructed to swallow 3 mL of cold water, which is injected into the oral cavity. The swallowing function of the pharynx was evaluated in five stages: (1) failure to drink because of choking and/or respiratory distress; (2) drinking without choking but with respiratory distress; (3) drinking with choking and/or hoarseness but without respiratory distress; (4) drinking without choking and without respiratory distress; and (5) the ability to perform two repetitive dry swallows within 30 s. The cutoff value was set at four points on the MWST [17].

The RSST score is measured as the number of times a patient could swallow saliva in 30 s [17].

For examination of vocal and respiratory functions, vocal function was measured using maximum phonation time (MPT), presence or absence of hoarseness, and hypernasality. To examine the respiratory function, the maximum expiratory flow rate (mL/min) was measured three times using a peak flow meter (Clement Clark, UK), and the mean value was calculated [19].

For evaluation of neck, facial, and tongue movements, the neck, face, and tongue movements were evaluated for abnormalities. Neck movements were evaluated for range of motion limitations in forward, backward, and lateral flexions. Facial movements were assessed for motor paralysis by instructing the patients to purse their lips, pull the corners of their mouth, close their lips, and inflate their cheeks. Tongue movements evaluation included lateral movements, tongue apex elevation, and back tongue elevation during /ka/ articulation.

### 2.3 Statistical Analyses

Statistical analyses were performed using JMP software (JMP Statistical Discovery LLC, USA, version 17). The Wilcoxon rank‐sum test was used for continuous variables. Pearson's chi‐square test was used to analyse categorical variables. These methods evaluated the association between characteristics of patients, oral functions, swallowing functions, and other functions and oral hypofunction. Spearman's rank correlation coefficient was used to correlate oral function tests and swallowing‐related criteria. Statistical significance levels were set at *p < 0.05, **p < 0.01.

## 3 Results

### 3.1 Characteristics of Patients at the Center for Dysphagia

The patient information is presented in Table 1. One hundred and ninety‐three patients were evaluated; finally, 183 patients (median age 69.0 years, 125 males and 58 females) were included in the analysis after excluding 10 patients with oral hypofunction who could not undergo a thorough examination for oral function. Continuous variables are presented as median, followed by the first (Q1) and third quartiles (Q3). Furthermore, the data for categorical variables are presented as the number of samples and percentages. Some subcategories had missing data.

**TABLE 1:** Characteristics of patients at the Center for Dysphagia.

	Total		Oral hypofunction patients		Non oral hypofunction patients		*p*
	*n* = 183		*n* = 154		*n* = 29		
	Median	(Q1, Q3)	Median	(Q1, Q3)	Median	(Q1, Q3)	
Age	69.0	(63.0, 76.0)	69.0	(64.0, 76.0)	69.0	(48.5, 77.5)	0.148
BMI	20.7	(18.5, 22.5)	20.7	(18.5, 22.4)	21.0	(18.5, 23.3)	0.843

		*n*	%	*n*	%	*n*	%	*p*
Sex	Male	125	68.3	108	59.0	17	9.3	0.222
	Female	58	31.7	46	25.1	12	6.6	
Primary disease	Head and neck tumour	85	46.6	71	38.3	14	7.7	0.503
	Degenerative disease	33	18.0	31	16.9	2	1.1	
	Muscle disease	23	12.7	18	9.8	5	2.7	
	Others	42	22.9	34	18.6	8	4.8	
History of a resection of the tongue or floor of the mouth	Absence	145	80.6	118	65.6	27	15.5	0.040*
	Presence	35	19.4	33	18.3	2	1.1	
	Missing	3						
History of a reconstruction of the tongue or floor of the mouth	Absence	145	81	120	67.0	25	14	0.224
	Presence	34	19	31	17.3	3	1.7	
	Missing	4						
Use of supplementary nutrition	Non	112	69.6	94	58.4	18	11.2	0.328
	Need	49	30.4	44	27.3	5	3.1	
	Missing	22						
Eichner's classification	A	71	39.0	57	31.3	14	7.7	0.027*
	B	67	36.8	54	29.7	13	7.1	
	C	44	24.2	43	23.6	1	0.6	
	Missing	1						
The need for removable dentures	No need	66	36.3	52	28.6	14	7.7	0.079
	In use	49	26.9	46	25.3	3	1.7	
	Necessary but unuse	67	36.8	55	30.2	12	6.6	
	Missing	1						

*Note:* Oral hypofunction is diagnosed when three or more of the seven assessment criteria are met. Eichner classification: Class A, with four occlusal support areas; Class B, with fewer occlusal support areas; and Class C, with no occlusal support areas. Wilcoxon rank‐sum test is used for continuous variables. Pearson's chi‐square test is used for categorical variables. Statistical significance levels are set at *p < 0.05.

Abbreviations: Q1, first quartile; Q3, third quartile.

The primary reasons of dysphagia, resulting in referral to the Center for Dysphagia at Dentistry, were head and neck tumours (85 patients, 46.4%), degenerative diseases (33 patients, 18.0%), and muscular diseases (23 patients, 12.7%). Among patients with head and neck tumours, 35 (41.2%) underwent resection of the tongue or floor of the mouth. The Eichner classification was 71 (39.0%) in Category A, 67 (36.8%) in Category B, and 44 (24.2%) in Category C. This category had one missing data. There were 67 (36.7%) patients who needed dentures but did not use them. There was a significant difference in the number of patients with oral hypofunction depending on whether they had a previous resection of the tongue or floor of the mouth (*p* = 0.040) and Eichner classification (*p* = 0.027).

### 3.2 Comparison of Oral Function With and Without Oral Hypofunction

The results of oral function examinations are shown in Table 2. Significant differences were observed between patients with and without oral hypofunction, such as in tongue coating (*p* = 0.001), number of remaining teeth (*p* = 0.007), ODK/ka/ (*p* = 0.047), tongue pressure (*p* = 0.033), mastication score (*p* = 0.004), and swallowing function questionnaire scores (Seirei swallowing questionnaire [15], *p* = 0.003; EAT‐10, *p* = 0.013).

**TABLE 2:** Comparison of oral functions with and without oral hypofunction.

		Total		Oral hypofunction patients		Non oral hypofunction patients		*p*
		Median	(Q1, Q3)	Median	(Q1, Q3)	Median	(Q1, Q3)	
TCI (%)		33.0	(17.0, 61.0)	39	(18.0, 66.0)	16.7	(11.0, 31.8)	0.001**
Oral dryness		28.9	(27.4, 31.2)	28.7	(27.3, 31.3)	29.7	(28.2, 31.2)	0.287
Number of remaining teeth		20.0	(9.0, 26.0)	19.0	(7.0, 26.0)	24.0	(20.8, 28.0)	0.007**
ODK/pa/(times/s)		4.6	(3.0, 6.0)	4.6	(3.1, 5.8)	5.4	(2.5, 6.4)	0.390
ODK/ta/(times/s)		4.6	(2.6, 5.8)	4.2	(2.6, 5.8)	5.6	(2.6, 6.2)	0.226
ODK/ka/(times/s)		4.0	(2.1, 5.4)	3.9	(2.1, 5.2)	5.2	(2.3, 6.3)	0.047*
Tongue pressure (kPa)		18.6	(7.4, 27.1)	17.9	(7.0, 26.6)	23.5	(14.0, 31.7)	0.033*
Masticatry performance (mg/dL)		107.0	(47.3, 169.8)	102.5	(45.0, 157.3)	159.0	(111.5, 193.8)	0.004**
Swallowing function (questionnaire score)	Seirei dysphagia screening questionnaire	3.0	(3.0, 6.0)	4.0	(3.0, 6.0)	0.5	(0.0, 1.8)	0.003**
	The EAT‐10	10.0	(1.3, 17.6)	10.0	(3.0, 22.0)	1.0	(0.0, 6.0)	0.013**

*Note:* Oral hypofunction is diagnosed when three or more of the seven assessment criteria are met. Statistical significance levels are set at *p < 0.05, **p < 0.01, respectively. Swallowing function is evaluated using the EAT‐10 or Seirei dysphagia screening questionnaire. The cut‐off value is set at 1 point on the Seirei dysphagia screening questionnaire and 3 points on the EAT‐10. Wilcoxon rank‐sum test is used for continuous variables. Pearson's chi‐square test is used to analyse categorical variables.

Abbreviations: Q1, first quartile; Q3, third quartile.

### 3.3 Comparison of Swallowing and Other Functional Examinations With and Without Oral Hypofunction

Th results of swallowing and other functional examinations (the maximum expiratory flow rate, MPT, OHI‐S score, hoarseness, hypernasality, and the abnormalities of neck, facial, and tongue movements) are presented in Table 3. Only the Hyodo–Komagane score differed significantly between patients with and without oral hypofunction (*p* = 0.003).

**TABLE 3:** Results of swallowing and other function.

	Total		Oral hypofunction patients		Non oral hypofunction patients		*p*
	Median	(Q1, Q3)	Median	(Q1, Q3)	Median	(Q1, Q3)	
The maximum expiratory flow rate (mL/min)	243.3	(156.7, 358.6)	243.3	(156.6, 355.8)	256.7	(143.3, 363.3)	0.921
MPT (s)	10.0	(7.0, 15.0)	10	(7.0, 14.0)	11.0	(7.8, 17.3)	0.564
OHI‐S score	0.5	(0, 1.5)	0.5	(0.0, 1.3)	0.3	(0, 1.7)	0.864
RSST (times/30 s)	3.0	(2.0, 5.0)	3	(2.0, 4.0)	4.0	(3.0, 5.5)	0.068
The Hyodo–Komagane score	2.0	(1.0, 4.0)	3.0	(1.0, 4.0)	1.0	(0.0, 3.0)	0.003**

		*n*	%	*n*	%	*n*	%	*p*
Hoarseness	Absence	103	58.6	85	48.6	18	10.3	0.370
	Presence	72	41.1	63	36.0	9	5.1	
	Missing	8						
Hypernasality	Absence	135	79.0	115	67.3	20	11.7	0.785
	Presence	36	21.1	30	17.5	6	3.5	
	Missing	12						
The abnormalities of neck movements	Absence	128	71.9	108	60.7	20	11.2	0.538
	Presence	50	28.1	44	24.7	6	3.4	
	Missing	5						
The abnormalities of facial movements	Absence	115	62.8	95	51.9	20	10.9	0.457
	Presence	68	37.2	59	32.2	9	4.9	
The abnormalities of tongue movements	Absence	107	58.5	87	47.5	20	10.9	0.211
	Presence	76	41.5	67	36.6	9	4.9	
MWST	Good	76	59.4	60	46.9	16	12.5	0.086
	Poor	52	40.6	47	36.7	5	3.9	
	Missing	55						

*Note:* Oral hypofunction is diagnosed when three or more of the seven assessment criteria are met. The cutoff value is set at 4 points on MWST. Wilcoxon rank‐sum test is used for continuous variables. Pearson's chi‐square test is used for categorical variables. Statistical significance levels are set at *p < 0.05, **p < 0.01, respectively.

Abbreviations: MWST, modified water swallowing test; Q1, first quartile; Q3, third quartile.

### 3.4 Association Between the Oral and Swallowing Functions

The correlations between the oral function tests and swallowing‐related functions are shown in Table 4. Positive correlations (*p* < 0.01) were observed between each articulation of the ODK, tongue pressure and ODK, mastication score and the number of remaining teeth, RSST and ODK, Hyodo–Komagane score and each swallowing questionnaire score. Correlations were observed between the Seirei swallowing questionnaire score and tongue pressure, and between the Hyodo–Komagane score and tongue pressure. Weak positive correlations were found between the number of remaining teeth and oral dryness, tongue pressure and oral dryness, mastication score and tongue pressure, RSST and oral dryness, RSST and ODK/pa/, Hyodo–Komagane score and ODK, and Hyodo–Komagane score and RSST.

**TABLE 4:** The relationship between the examination of the oral functions and swallowing‐related criteria.

	TCI	OHI‐S	Oral dryness	Number of remaining teeth	ODK					Swallowing function			The Hyodo score
					/pa/	/ta/	/ka/	Tongue pressure	Masticatory performance	The Seirei dysphagia screening questionnaire	The EAT‐10	RSST	
TCI	—												
OHI‐S	0.040	—											
Oral dryness	0.056	0.092	—										
Number of remaining teeth	−0.149	−0.095	0.164*	—									
ODK													
/pa/	0.072	−0.140	−0.15	0.066	—								
/ta/	0.125	−0.116	−0.061	0.090	0.809**	—							
/ka/	0.079	−0.102	−0.075	0.083	0.817**	0.898**	—						
Tongue pressure	0.082	−0.034	0.152*	0.110	0.320**	0.426**	0.404**	—					
Masticatory performance	−0.213*	−0.141	0.109	0.443**	0.125	0.112	0.065	0.246*	—				
Swallowing function													
The Seirei dysphagia screening questionnaire	−0.089	0.087	0.051	0.024	−0.210	−0.312*	−0.330*	−0.444**	0.029	—			
The EAT‐10	−0.051	−0.187	0.114	0.093	−0.069	−0.153	−0.131	−0.221*	−0.173	0.783	—		
RSST	0.111	0.007	0.180*	0.141	0.172*	0.220**	0.275**	0.291**	0.137	−0.100	−0.146	—	
The Hyodo–Komagane score	0.123	0.053	−0.101	−0.046	−0.192*	−0.183*	−0.192*	−0.330**	0.075	0.468**	0.289**	−0.155*	—

*Note:* Swallowing function is evaluated by the EAT‐10 or Seirei dysphagia screening questionnaire. Spearman's rank correlation coefficient is used to determine the correlation between oral function tests and swallowing‐related criteria. Statistical significance levels are set at *p < 0.05 and **p < 0.01.

Abbreviations: EAT‐10, 10‐item Eating Assessment Tool; MWST, modified water swallowing test; ODK, oral diadochokinesis; OHI‐S, simplified oral hygiene index; RSST, repetitive saliva swallowing test.

## 4 Discussion

This study evaluated oral hypofunction, the most common symptom of oral frailty, and investigated the association between oral and swallowing functions. We focused on patients with dysphagia, including those who were suspected of dysphagia due to head and neck tumours, degenerative diseases, and muscular diseases. This study also investigated the oral functional parameters that were closely related to swallowing functions to propose effective practical methods of dental intervention.

The prevalence of oral hypofunction, a condition characterised by reduced oral function, was 84.2% among patients who visited the Center for Dysphagia. This rate is very high compared with previous reports (35.9%–62.9%) of nursing home residents [4], community residents [20], university hospital outpatients [21], and dental patients. This can be attributed to the fact that this center was established as a specialised center for treatment of swallowing in a large population of patients with dysphagia including head and neck tumours, degenerative diseases and muscular diseases. For instance, patients who underwent tongue or oral floor resection showed a significantly higher prevalence of oral hypofunction than those who did not. Surgical resection of oral cancer causes functional impairments in mastication, swallowing, pronunciation, and articulation [22, 23, 24]. Therefore, the prevalence of oral hypofunction in patients who have undergone surgical resection may have increased.

A strong correlation between the oral and swallowing functions was confirmed in this study; therefore, there may be interconnectedness of these aspects in dysphagia. Differences in the number of occlusal support areas and remaining teeth were observed, depending on the presence or absence of oral hypofunction. Additionally, the number of remaining teeth showed a correlation with masticatory function values, consistent with previous reports [21]. However, there was no relationship between the need for dentures and the presence or absence of oral hypofunction, suggesting that other factors may play a role. Furthermore, regarding oral hygiene, there was an association between tongue coating and the presence of oral hypofunction, but not between OHI‐S and the presence of oral hypofunction. This might be due to the comprehensive oral care provided as a part of standard care to all hospitalised patients with dysphagia by dental hygienists, nurses, and other staff.

Oral dryness and ODK (/pa/ and /ta/) did not differ significantly between patients with and without oral hypofunction. Generally, oral dryness and ODK (/pa/ and /ta/) were thought to be related in healthy old adults [25]. However, since patients' primary diseases of head and neck tumours, degenerative diseases, and muscular diseases accounted for 58%, these patients are likely to have characteristics of oral dryness and lower ODK (/pa/ and /ta/). Therefore, these parameters were not associated with the diagnosis of oral hypofunction.

In the evaluation of the function of swallowing, significant differences were found only in Hyodo–Komagane score between patients with and without oral hypofunction. When the association between four evaluations of the Hyodo–Komagane score and the presence of oral hypofunction was analysed, there were significant differences only in the pharyngeal clearance score after swallowing (*p* = 0.0085). In addition, there were significant differences between tongue pressure and pharyngeal clearance after swallowing (*p* < 0.0001, Table 2). This may be because applying palatal augmentation prosthesis to postoperative patients with tongue cancer had improved oral function, including tongue pressure, and decreased hypopharyngeal residuals [26]. It was suggested that patients with oral hypofunction who have decreased tongue pressure may have hypopharyngeal residuals.

The Hyodo–Komagane score was correlated with the ODK, tongue pressure, and swallowing questionnaire. The correlation of the ODK and tongue pressure may be because 41.5% of the population in this study had limitations in tongue movement. The Seirei dysphagia screening questionnaire and EAT‐10 being highly sensitive questionnaires for assessing dysphagia [15, 27], significant correlations may be observed between Hyodo–Komagane scores and the questionnaires.

In general, during masticatory movement, the tongue moves food onto the occlusal surfaces of the teeth and mixes the food segment and saliva to ensure proper bolus formation. The bolus can then be easily swallowed. Thus, normal smooth pharyngeal function requires proper tongue movements such as tongue pressure and ODK. Therefore, the results of this study suggest that the oral dryness score, ODK, and tongue pressure related to masticatory activity and/or swallowing movements are associated with oral hypofunction and dysphagia. However, this study has some limitations such as potential biases because participants were recruited at only Tohoku University Hospital.

The Center for Dysphagia is a multi‐professional team that includes the departments of Otolaryngology, Head and Neck Surgery, Physical Medicine and Rehabilitation, Dentistry, Pharmacy, Nursing, Dietitian Office, and Dental Hygiene Office. This collaborative approach enables the team to measure not only oral but also swallowing function simultaneously and ensures that patients with dysphagia receive holistic care that addresses their medical, physical, and nutritional requirements. The practical implications of our findings are that in patients with dry mouth, poor ODK, or low tongue pressure, their ability to swallow might improve upon being admitted to a hospital and if they begin early rehabilitation with a therapist or dental professional. Specifically, this early intervention might reduce the amount of food or liquid left in the hypopharyngeal space after swallowing.

This study has some limitations such as cross‐sectional study, single‐center setting, and selection bias. The nature of this study resulted in selection bias. It is possible that the patients who stayed in hospital for a short time, were in serious state for a long time, or died cannot be recruited. Tohoku university hospital is an acute hospital and the biggest hospital in the northern region of Japan. Thus, this single‐center study may have included more serious patients. A potential limitation is the structural overlap between the diagnosis of oral hypofunction and the assessment of swallowing function. Because the diagnostic criteria for oral hypofunction already include swallowing questionnaires (EAT‐10 or Seirei questionnaire), the two variables are not entirely independent. Consequently, the association between them may be mutually affected, potentially leading to inflated correlation.

## 5 Conclusions

A high prevalence of oral hypofunction was observed in approximately 60% of patients who had head and neck tumours, degenerative diseases, and muscular diseases visiting the Center for Dysphagia at the Division of Dentistry. Additionally, 41.5% of patients had limited tongue movement, and this may have linked to correlation between ODK and Hyodo–Komagane scores. These results suggest that oral dryness score, ODK, and tongue pressure are associated with oral hypofunction and dysphagia. Importantly, it suggested that patients with hypofunction and decreased tongue pressure might have hypopharyngeal residuals. This insight could affect the treatment and care of patients with dysphagia in the dental field.

Clinical implications of our findings are as follows: the medical‐dental cooperation in an acute hospital allows for the simultaneous assessment of oral and swallowing functions, which is essential for patients with dysphagia. The results of the assessment suggest a potential to contribute to the practice of safe oral intake and appropriate rehabilitation, and the use of less invasive oral function assessment as a screening test.

## Author Contributions

**Ryo Tagaino:** collected the clinical data from medical records, drafted the manuscript and prepared the tables and figures. **Kana Saijo:** collected the clinical data from medical records. **Naru Shiraishi:** conceived and designed the study, performed descriptive statistical analysis, contributed to data interpretation and critical discussion, critically reviewed and revised the manuscript for important intellectual content. **Takamasa Komiyama:** collected the clinical data from medical records. **Kuniyuki Izumita:** collected the clinical data from medical records. **Takuma Hisaoka:** collected the clinical data from medical records. **Ai Hirano:** collected the clinical data from medical records. **Jun Ota:** collected the clinical data from medical records. **Yukio Katori:** critically reviewed and revised the manuscript for important intellectual content. **Toru Ogawa:** critically reviewed and revised the manuscript for important intellectual content. **Keiichi Sasaki:** critically reviewed and revised the manuscript for important intellectual content. **Nobuhiro Yoda:** critically reviewed and revised the manuscript for important intellectual content. **Hiroshi Egusa:** critically reviewed and revised the manuscript for important intellectual content. **Shigeto Koyama:** critically reviewed and revised the manuscript for important intellectual content, supervised the study design, data analysis, and manuscript preparation. All authors read and approved the final manuscript.

## Funding

This work was supported in part by Grants‐in Aid for Scientific Research (KAKENHI) (Grant Number 24K19935, 23K092911 and 23K09291).

## Ethics Statement

This study was approved by the Ethics Committee of Tohoku University Graduate School of Dentistry (approval number: 38677, committee number: 11000400).

## Conflicts of Interest

The authors declare no conflicts of interest.

## Data Availability

The data that support the findings of this study are available from the corresponding author upon reasonable request.
